# 

**Published:** 1881-08

**Authors:** 


### CONTENTS.

*ORIGINAL:*

PAGE.

Unproved Remedies. P. P. Wells, M. D., Brooklyn, N. Y., . . . 357
Missouri Institute, . . . . . . . . . . . . 362
Fatal Errors. Ad. Lippe, M. D., Philadelphia, . . . . . . 363
The Hering Memorial Volume. E. W. Berridge, M. D., London, . . 365
Fluxion Dilutions. Eldridge C. Price, M. D., Baltimore, Md., . . 366
The Penn Medical University; An open letter from "The Historian," 370
Hæmorrhoids and their Treatment. Dr. A. Chargé, Paris, France, . 372

*CLINICAL BUREAU:*

*Picric Acid*—Ophthalmia; *Natr. mur.*—Neuralgia and Cough; *Mang. carb.*—Colic; *Spongia*—Chronic Winter Cough. E. W. Berridge, M. D., London, . . . . . . . . . . . . 376

Bell—Croup. W. M. James, M. D., Philadelphia, . . . . . 385

*Cina*—Rheumatism. W. A. Hawley, M. D., Syracuse, N. Y., . . . 386

*Eupatorium purp.*—Intermittent Fever. C. W. Butler, M. D., Montclair, N. J., . . . . . . . . . . . . . . 387

Periscope, . . . . . . . . . . . . . . 390

*BOOK NOTICES AND REVIEWS:*

Homœopathic Therapeutics of Diarrhœa, Etc. Drs. J. B. Bell and W. T. Laird, . . . . . . . . . . . . . . 396

Treatise on Diseases of Infants and Children. W. A. Edmonds, M. D., 396

Diseases of the Nervous System. Charles P. Hart, M. D., . . . 397

Treatise on Diphtheria. A. McNeil, M. D., . . . . . . . 398

Errata, . . . . . . . . . . . . . . . 400

